# Outcomes of Endoscopic Resection in Pediatric Skull Base Chordoma: A Systematic Review

**DOI:** 10.7759/cureus.41487

**Published:** 2023-07-07

**Authors:** Thamer H Alsharif, Amin G Gronfula, Lamees H Alghdali, Mayasim Hejazi, Abdulkarim Alanazi, Sahal M Wali, Mohammed Alyousef

**Affiliations:** 1 Neurosurgery, Royal College of Surgeons in Ireland, Dublin, IRL; 2 Orthopaedic Surgery, Royal College of Surgeons in Ireland, Dublin, IRL; 3 Internal Medicine, Royal College of Surgeons in Ireland, Dublin, IRL; 4 Emergency Medicine, Royal College of Surgeons in Ireland, Dublin, IRL; 5 Radiology, Royal College of Surgeons in Ireland, Dublin, IRL; 6 Surgery, King Abdulaziz University Hospital, Jeddah, SAU

**Keywords:** children, endoscopic resection, chordoma, skull base, pediatric

## Abstract

The endoscopic approach has been recommended as a primary option for treating chordomas, and it is associated with better resection rates and fewer surgical complications than transcranial surgery. This review aimed to assess the long-term consequences and evidence in the current literature regarding the endoscopic approach's efficacy in treating skull-base chordoma in children. A systematic review was conducted based on the PubMed, Web of Science, and EMBASE databases to examine the clinical outcomes of endoscopic endonasal surgery for pediatric skull base chordoma tumors. The review included studies published in English that employed specific research designs and reported on pediatric patients with skull base chordoma. Of the 268 studies initially considered, 25 met our eligibility criteria and were included in the final analysis. The average age of the patients was 11.5 years, with approximately equal number of males and females. The endoscopic endonasal approach (EEA) was the most commonly used modality. Gross total resection (GTR) was achieved in 62.7% of patients, while 18.09% had a subtotal resection (STR), and 13.83% had near-total resection only. Most patients showed significant to moderate improvement from their baseline condition and had no recurrence during their follow-up. Our findings further endorse that the endoscopic approach is a viable primary treatment option for pediatric skull base chordoma.

## Introduction and background

Pediatric skull base chordoma is a low-grade and rare neoplasm originating from the notochordal remnants and arising in the spinal axis from the skull base to the sacrum [1,2]. Chordomas are considered to have aggressive behavior marked by local invasion. However, their behavior can be described as tumor progression that is “clinically malignant” because of the diffusely infiltrative growth pattern, high recurrence rates, and tumor-related mortality rate. Furthermore, the tumor also spreads to the surrounding areas of the skull base, with an incidence rate of 0.08 in 100,000 population. Children affected by chordoma may experience a more aggressive form of the disease than adults. They often have non-specific symptoms such as double vision, headaches, vertigo, and visual difficulties. Mild deficits may go unnoticed until they start to affect daily activities, leading to a delay in diagnosis. By this point, the tumor may have already spread to multiple compartments [3].

Chordomas are one of the most difficult cranial base tumors to treat due to their invasiveness, high recurrence, and mortality rate [4]. Surgical resection is the best treatment modality, which could be followed by proton-beam radiotherapy (PBRT) for microscopic residual [5]. In recent years, the endoscopic endonasal approach (EEA) has been the first-line treatment method with a higher resection rate and a lower surgical complication rate than transcranial surgery for managing chordomas [2,6]. The EEA provides a safe and reliable method for resecting chordomas and offers benefits in terms of expansive tumor exposure and direct visualization, particularly for tumors that infiltrate the cavernous sinus, suprasellar region, or clivus [7,8].

Endoscopic skull base surgery has minimal anatomical limitations, with most structures being resectable or mobilizable. However, challenges exist regarding the internal carotid artery, cerebral involvement, optic nerve invasion, and lesions extending beyond the reach of the endonasal approach [9-11]. Other limitations pertain to the specialized instruments and precise endoscopes required. The cost of equipment, including disposable items, should be considered, especially when compared to the expenses associated with conventional open surgery [12].

## Review

Methods

​​​*Data Sources and Search Strategy *

We conducted this review by following the Preferred Reporting Items for Systematic Reviews and Meta-Analyses (PRISMA) guidelines. An electronic search on the following databases was conducted for articles from inception until March 2022: PubMed, EMBASE, Web of Science, and the Cochrane Library. 

Eligibility Criteria

Inclusion criteria: studies that involved endoscopic endonasal surgery for skull base tumors in pediatric patients; studies that reported clinical outcomes such as neurological improvement, pain relief, and quality of life postoperatively; studies that included pediatric patients aged 18 years or younger with skull base chordoma; randomized controlled trials (RCTs), quasi-randomized trials, prospective observational studies, case reports or retrospective studies, and studies published in English.

Exclusion Criteria: studies that included adult patients with skull base chordoma; studies about endoscopic endonasal surgery in adults; systematic reviews or conference abstracts; and studies not published in English.

Based on the inclusion and exclusion criteria, two reviewers independently screened studies by reading titles and abstracts and removed duplicates. Finally, they confirmed the eligible studies for this systematic review, and any disagreement between them was resolved by a third reviewer. 

Data Extraction and Quality Assessment 

Two reviewers (LA & MH) independently conducted literature screening, data extraction, and cross-checking. The information extracted was entered into a Google Sheet, and it included the following aspects: first author, year of publication and journal, country of origin, title, number of cases, patient age, gender, symptoms, diagnosis, surgery, the extent of resection, postop data, early or late complications, and follow-up details. A third reviewer (TA) resolved any disagreements between the two primary reviewers. The statistical analysis was performed using Microsoft Excel version 16.72.

Results

Study Characteristics

**Figure 1:**
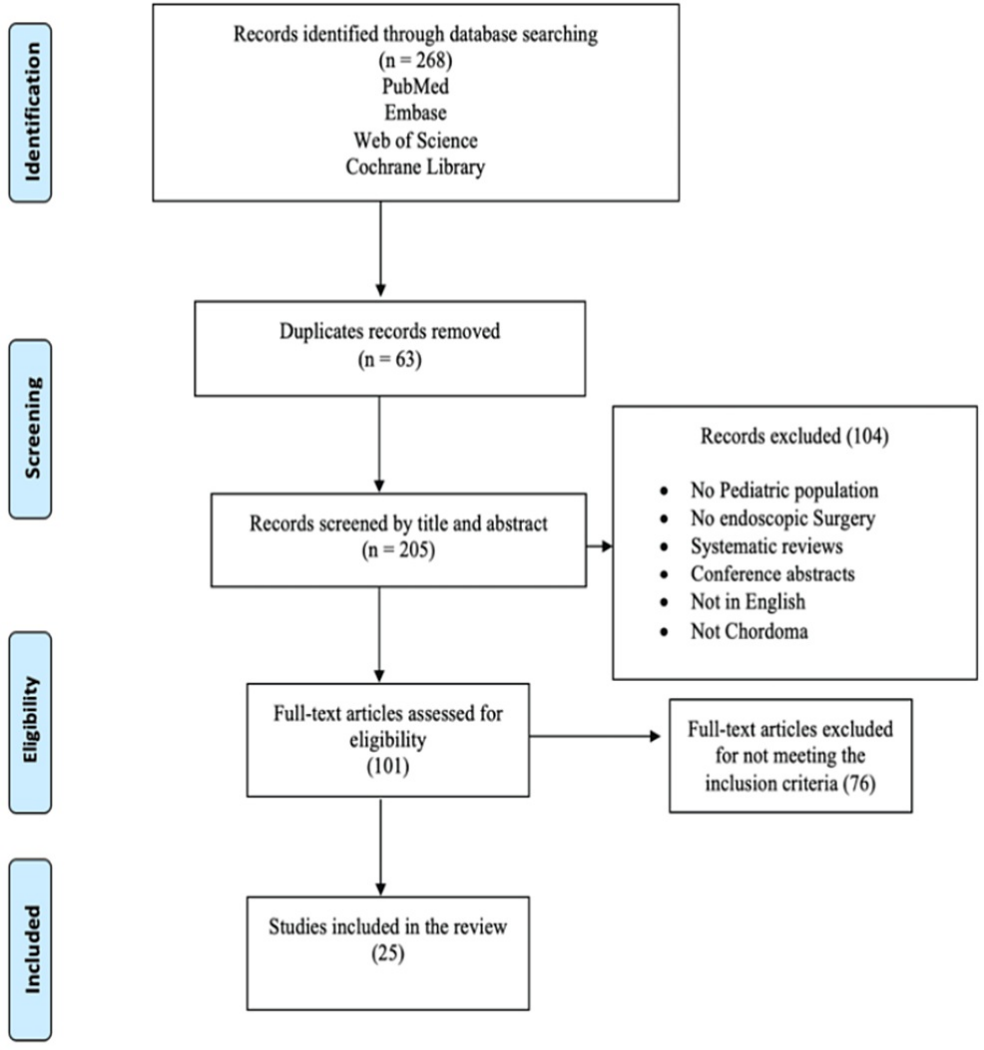
PRISMA flow diagram depicting the selection of articles PRISMA: Preferred Reporting Items for Systematic Reviews and Meta-Analyses

The search encompassed four databases: PubMed, Web of Science, Embase, and Cochrane Library, yielding 268 articles. After removing duplicate articles, 205 remained for initial screening based on title and abstract, which led to the subsequent exclusion of 104 more articles. The remaining 101 articles underwent full-text screening, and we ultimately included 25 articles in the qualitative analysis. Of them, 20 were randomized studies, and five were case reports or series. These articles were from the Middle East, Asia, Europe, the UK, and the USA. The PRISMA diagram depicting the selection of articles is shown in Figure 1.

Study Population 

The studies included in the systematic review involved a total of 94 patients. The average age of the patients was 11.5 years (range: 1-18 years). The male-to-female ratio was 1.35/1. A retrospective cohort study design was used in 20 studies, while five were case reports. A total of 103 procedures were performed, with EEA accounting for the majority of them. The extent of resection was reported in 94 patients, with gross total resection (GTR) performed in 59 patients (62.77%). The average follow-up duration in 62 patients was 32 months (3-158 months), and 16 had a recurrence (Table 1).

**Table 1:** Characteristics of the selected studies

Characteristic	Value
Number of studies included	25
Study design, n (%)	
Retrospective cohort	20 (80)
Case reports	5 (20)
Number of patients	94
Age in years, average (range)	11.5 (1—18)
Male, n (%)	46 (48.94)
Female, n (%)	35 (37.23)
Not available, n (%)	13 (13.83)
Total number of procedures	103
Type of procedure, n (%)	
Endoscopic endonasal approach	88 (85.44)
Endoscopic retrosigmoid approach	8 (7.7)
Endoscopic transnasal approach	3 (2.91)
Endoscopic transoral approach	2 (1.94)
Endoscopic transcondylar approach	1 (0.97)
Endoscopic suboccipital approach	1 (0.97)
Extent of resection, n (%)	
Gross total resection	59 (62.77)
Subtotal resection	17 (18.09)
Near-total resection	13 (13.83)
Partial resection	2 (2.13)
Not available	3 (3.19)
Follow-up duration in months (in 62 patients), average (range)	32 (3–158)
Total number of recurrence	16
Time to recurrence in months, average (range)	7.26 (1–27)

Location of the Tumors 

As illustrated in Table 2 below, most patients had tumors in the unspecified skull base region (43/58), while 39 patients had tumors located specifically in the clivus region. The remaining patients had tumors in various locations, including the suprasellar, cavernous sinus, and prepontine intradural.

**Table 2:** Location of the tumors

Site of the tumor	Number of patients
Clivus	39
Suprasellar	3
Cavernous sinus	1
Prepontine intradural	1
Skull base, unspecified	43
Posterior fossa, unspecified	11

Clinical Presentation 

The most common symptoms and signs of skull base tumors were headaches (n=31 patients), followed by cranial nerve (CN) palsy in 30 patients, and visual changes in 23 patients. Endocrinopathy was reported in five patients, and other less common symptoms included difficulty swallowing, upper limb weakness, neck pain, torticollis, obstructive sleep apnea, nasal congestion, tetraparesis, anosmia, dysarthria, drooling, facial numbness, facial weakness, fever, and brainstem dysfunction (unspecified). Table 3 shows a breakdown of all signs and symptoms.

**Table 3:** Symptoms and signs among the patients

Symptoms and signs	Number of patients
Headache	31
Cranial nerve palsy	30
Visual changes	23
Endocrinopathy	5
Difficulty swallowing	3
Upper limb weakness	2
Neck pain	2
Torticollis	2
Obstructive sleep apnea	2
Nasal congestion	2
Tetraparesis	1
Anosmia	1
Dysarthria	1
Drooling	1
Facial numbness	1
Facial weakness	1

Complications 

As shown in Table 4, the most common early complication (<6 weeks) was cerebrospinal fluid (CSF) leak (n=20 patients), followed by CN palsy in five patients and vascular injury in three patients. The most common late complication (>6 weeks) was CN palsy in one patient and meningitis and delayed diabetes insipidus in one patient each. The average time to recurrence was 7.26 months (range: 1-27 months). The combined results showed that endoscopic skull base surgery for chordoma in pediatric patients is a safe and effective method for treating skull base tumors, with a high rate of GTR. However, this procedure is associated with a risk of complications, particularly CSF leak and CN palsy. The most common symptoms of skull base tumors are headache, CN palsy, and visual changes. Further studies with larger sample sizes and longer follow-up periods are needed to confirm these findings and identify strategies for reducing the risk of complications.

**Table 4:** Complications

Complications	Number of patients
Early complications (<6 weeks)	
Cerebrospinal fluid leak	20
Cranial nerve palsy	5
Vascular injury	3
Headache	1
Infection/sepsis	1
Intraoperative hemorrhage	1
Transient quadriplegia	1
Meningitis	1
Epistaxis	1
Occipital cervical instability	1
Epidural hematoma	1
Nausea/vomiting	1
Late complications (>6 weeks)	
Cranial nerve palsy	1
Meningitis	1
Delayed diabetes insipidus	1

Discussion 

Skull base chordoma is a low-grade and rare neoplasm that can occur in the pediatric population. When it occurs, it requires skilled management to prevent significant morbidity [13]. In recent years, there has been a surge in the use of the EEA for the surgical management of pediatric skull base diseases. This may be due to the emergence of dedicated skull base surgeons, early and increased exposure to EEA techniques during training, and increased collaboration between pediatric neurosurgeons and otolaryngologists [14]. However, there are no studies in the current literature that examines the operative outcomes of EEA in skull base chordoma in the pediatric population.

EEA is uniquely advantageous for the resection of midline clival lesions such as chordoma. The direct, midline approach allows for visualization, definition, and resection of margins bilaterally with ease while allowing for resection down past the foramen magnum into the upper cervical spine when needed. Superiorly and posteriorly, the origin of the tumor tends to displace critical neurovascular structures favorably, whereas open cranial approaches tend to result in encountering these structures early on, forcing surgeons to work around and manipulate them for a greater portion of the resection [15].

Recent cohort studies have reported higher rates of GTR with EEA, with a rate of 62.77% in the examined case series. Subtotal resection (STR) was achieved in 18% of cases [16,17]. The most common presenting symptoms were headache and CN palsy, while the common complication reported following EEA was CSF leak postoperatively. Overall, the literature shows the safety and efficacy of EEA techniques in the pediatric population for managing skull base chordoma [18,19].

The findings of this systematic review suggest that endoscopic skull base surgery is a feasible and safe approach for managing skull base chordoma, with acceptable rates of complications and recurrence. However, it is essential to note that most of the studies included in this review were retrospective cohort studies, and only a small number of patients were included. Therefore, the results of this review should be interpreted with caution, and further research is needed to confirm these findings. Moreover, it is essential to consider the limitations of the study design when interpreting the results. For example, retrospective studies may have incomplete or inaccurate data, and there may be selection bias due to the non-randomized nature of the study design. Furthermore, case reports may be susceptible to publication bias, as only the most interesting or unique cases are typically reported in the literature.

Despite these limitations, the results of this systematic review are encouraging and provide important insights into the safety and efficacy of endoscopic skull base surgery for the management of chordoma located in the skull base. The findings suggest that this approach may be a better alternative to traditional open surgery, particularly for chordomas located in the skull base in the pediatric population.

## Conclusions

Based on our findings, endoscopic skull base surgery is a promising technique for treating skull base chordoma in the pediatric population, with a high rate of GTR and acceptable rates of complications and recurrence. However, more extensive prospective studies are needed to confirm these findings and determine the long-term outcomes of this approach. Furthermore, ongoing research is needed to identify strategies for minimizing the risk of complications and improving the efficacy of this approach for managing such tumors in children.
